# Correction: Neuropilin-1 controls vascular permeability through juxtacrine regulation of endothelial adherens junctions

**DOI:** 10.1007/s10456-024-09968-y

**Published:** 2025-02-04

**Authors:** Sagnik Pal, Yangyang Su, Emmanuel Nwadozi, Lena Claesson-Welsh, Mark Richards

**Affiliations:** Department of Immunology, Genetics and Pathology, Beijer and Science for Life Laboratories, Uppsala University, Uppsala, Sweden

**Correction to: Angiogenesis (2024) 28:7** 10.1007/s10456-024-09963-3

In the original published article, Fig. 2F was processed incorrectly. The incorrect and correct version of Fig. 2 were provided in this correction.

The original article has been corrected.

**Incorrect version:**

**Fig. 2:**
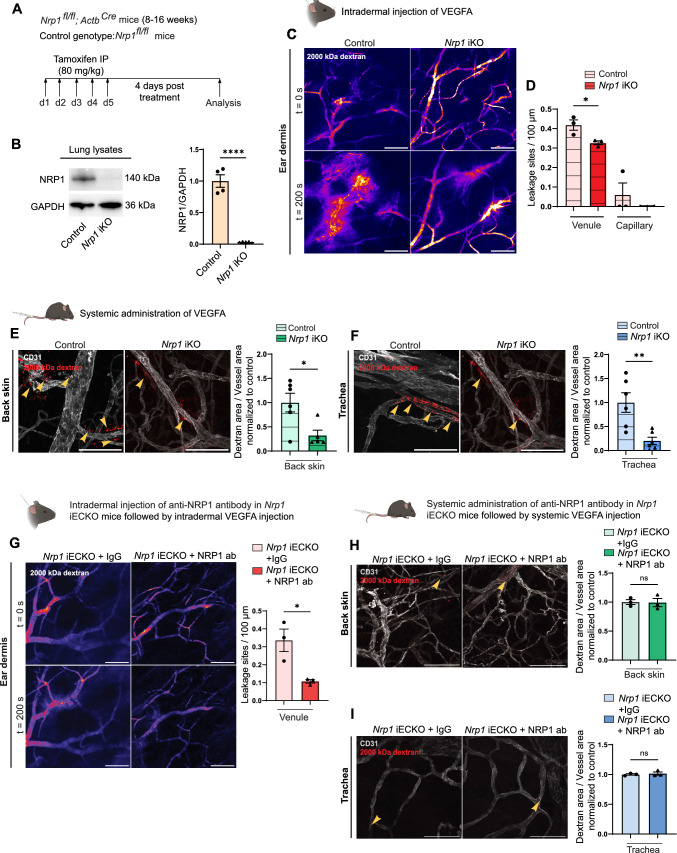
Global loss of NRP1 reduces VEGFA-mediated vascular permeability. **A** Schematic illustration of experimental design to induce recombination in *Nrp1*^*fl/fl*^*; Actb*^*Cre*^ (*Nrp1* iKO) mice. *Nrp1*^*fl/fl*^ were used as control. **B** Western blot and quantification of NRP1 protein levels in lung lysates from tamoxifen-treated control and *Nrp1* iKO mice (n ≥ 3 mice). **C** Representative images showing leakage of 2000 kDa FITC dextran (Pseudo-colour) in response to intradermal VEGFA injection in the ear of control and *Nrp1* iKO mice. **D** Leakage sites per vessel length in response to intradermal VEGFA stimulation in the ear skin of control and *Nrp1* iKO mice. N = 3 mice, two or more acquisitions/mouse. **E** and **F** Leakage of fixable 2000 kDa FITC dextran in back skin (**E**) and trachea (**F**) after systemic administration of VEGFA in control and *Nrp1* iKO mice. Left, representative images. Right, quantification of tracer leakage area/vessel area (n ≥ 5 mice, 3 or more fields of view/mouse). **G** VEGFA-induced leakage in the ear skin of *Nrp1* iECKO mice treated intradermally with isotype control or NRP1-VEGFA blocking antibody. Left, representative images. Right, quantification of leakage sites per 100 µm of vessel length (n = 3 mice, ≥ 2 acquisitions/mouse). **H** and **I** VEGFA-induced leakage of 2000 kDa dextran in back skin (**H**) and trachea (**I**) of *Nrp1* iECKO mice treated systemically with isotype control or NRP1-VEGFA blocking antibody. Left, representative images. Right, quantification of tracer area/vessel area (n = 3 mice). Error bars; mean ± SEM. Statistical significance: Two-tailed unpaired Student’s t-test. Scale bar: 100 μm

**Correct version:**

**Fig. 2:**
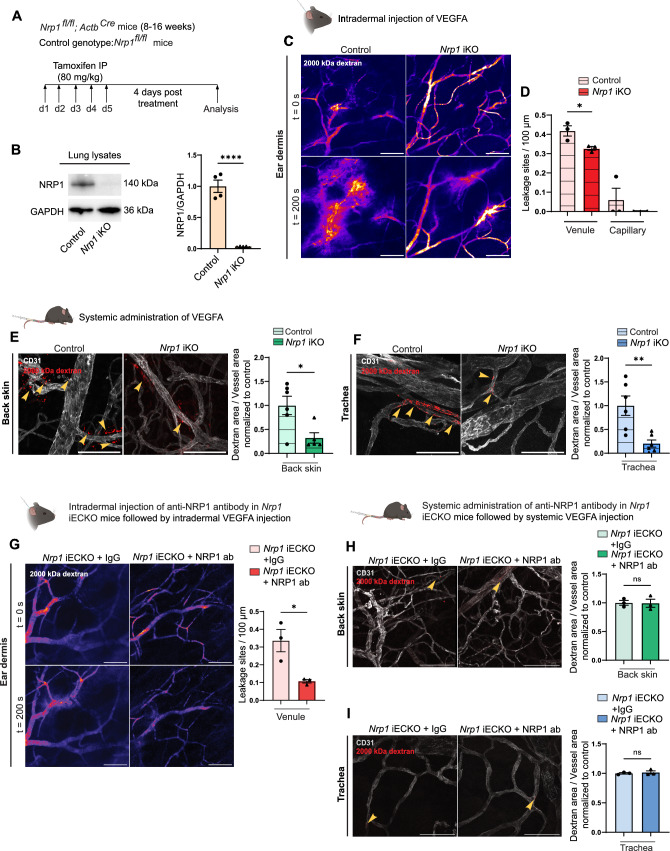
Global loss of NRP1 reduces VEGFA-mediated vascular permeability. **A** Schematic illustration of experimental design to induce recombination in *Nrp1*^*fl/fl*^*; Actb*^*Cre*^ (*Nrp1* iKO) mice. *Nrp1*^*fl/fl*^ were used as control. **B** Western blot and quantification of NRP1 protein levels in lung lysates from tamoxifen-treated control and *Nrp1* iKO mice (n ≥ 3 mice). **C** Representative images showing leakage of 2000 kDa FITC dextran (Pseudo-colour) in response to intradermal VEGFA injection in the ear of control and *Nrp1* iKO mice. **D** Leakage sites per vessel length in response to intradermal VEGFA stimulation in the ear skin of control and *Nrp1* iKO mice. N = 3 mice, two or more acquisitions/mouse. **E** and **F** Leakage of fixable 2000 kDa FITC dextran in back skin (**E**) and trachea (**F**) after systemic administration of VEGFA in control and *Nrp1* iKO mice. Left, representative images. Right, quantification of tracer leakage area/vessel area (n ≥ 5 mice, 3 or more fields of view/mouse). **G** VEGFA-induced leakage in the ear skin of *Nrp1* iECKO mice treated intradermally with isotype control or NRP1-VEGFA blocking antibody. Left, representative images. Right, quantification of leakage sites per 100 µm of vessel length (n = 3 mice, ≥ 2 acquisitions/mouse). **H** and **I** VEGFA-induced leakage of 2000 kDa dextran in back skin (**H**) and trachea (**I**) of *Nrp1* iECKO mice treated systemically with isotype control or NRP1-VEGFA blocking antibody. Left, representative images. Right, quantification of tracer area/vessel area (n = 3 mice). Error bars; mean ± SEM. Statistical significance: Two-tailed unpaired Student’s t-test. Scale bar: 100 μm

