# Supplementary material for: Neuropilin-1 controls vascular permeability through juxtacrine regulation of endothelial adherens junctions
Source: Angiogenesis. 2024 Dec 12;28(1):7. doi: 10.1007/s10456-024-09963-3 (PMC11638295; doi:10.1007/s10456-024-09963-3)
Supplement: Supplementary file 1 — Supplementary file1 (DOCX 15 kb) [file 10456_2024_9963_MOESM1_ESM.docx]

**Supplementary Figure 1. NRP1 expression in the ear dermis and back skin of adult mice**

**(A)** Western blot and quantification of NRP1 expression using lung lysates from tamoxifen-treated control *Nrp1^fl/fl^* and *Nrp1* iECKO mice (n = 4).

**(B,C)** Whole mount immunostainings showing NRP1 expression in ear dermis **(B)** and back skin **(C)** of control *Nrp1^fl/fl^*, *Nrp1* iECKO and *Nrp1* iKO mice. Scale bar: 50 μm.

**(D,E)** Images, left, and quantification, right, of vascular and perivascular NRP1 expression in ear skin and back skin of control *Nrp1^fl/lf^* and *Nrp1* iECKO mice. Scale bar: 20 μm.

Error bars; mean ± SEM. Statistical significance used: Two-tailed unpaired Student’s t-test.

**Supplementary Figure 2. Organotypic assessment of VEGFA-induced leakage**

**(A**) Basal permeability of 10 kDa dextran in kidney, back skin, ear dermis, skeletal muscle and heart in control *Nrp1^fl/fl^* and *Nrp1* iECKO mice. Extravasated dextran was measured following extraction from perfused tissues. (n ≥ 8 mice).

**(B,C)** Leakage of 2000 kDa **(B)** and 70 kDa **(C)** dextran after systemic administration of only dextran (PBS) or dextran plus VEGFA in back skin, trachea, kidney, skeletal muscle and heart of wild-type C57Bl/6 mice (n ≥ 3 mice).

**(D,E)** VEGFA-mediated leakage of 70 kDa **(D)** and 2000 kDa **(E)** dextran in kidney, skeletal muscle and heart in control *Nrp1^fl/fl^* and *Nrp1* iECKO mice. Extravasated dextran was measured following extraction from perfused tissues. (n ≥ 8 mice).

Error bars; mean ± SEM. Statistical significance used: Two-tailed unpaired Student’s t-test.

**Supplementary Figure 3. Perivascular NRP1 modifies PLCγ phosphorylation**

**(A-B)** Phosphorylation of PLCγ in response to intradermal PBS or VEGFA injections in the ear dermis of control *Nrp1^fl/fl^* and *Nrp1* iECKO **(A)** or *Nrp1* iKO **(B)** mice. Left, representative images. Right, quantification of phosphorylated PLCγ area per total VEC area normalized to PBS control in the ear dermis of control *Nrp1^fl/fl^* and *Nrp1* iECKO or *Nrp1* iKO mice. n ≥ 3 mice, two or more fields of view / mouse.

**(C-D)** Phosphorylation of PLCγ in response to intradermal PBS or VEGFA injections in the back skin of control *Nrp1^fl/fl^* and *Nrp1* iECKO **(C)** or *Nrp1* iKO **(D)** mice. Left, representative images. Right, quantification of phosphorylated PLCγ area per total VEC area normalized to PBS control in the back skin of control *Nrp1^fl/fl^* and *Nrp1* iECKO or *Nrp1* iKO mice. n ≥ 3 mice, two or more fields of view/ mouse.

Error bars; mean ± SEM. Statistical significance: Two-way ANOVA. Scale bar: 50 μm.

**Video 1: VEGFA-induced leakage in control *Nrp1*^fl/fl^, *Cdh5* Cre-negative littermate mice**

Extravasation of circulating 2000 kDa FITC Dextran (pseudocolor) in control *Nrp1*^fl/fl^, *Cdh5* Cre-negative littermate mice after intradermal injection of VEGFA in the ear dermis. Video corresponds to stills in Figure 1D.

**Video 2: VEGFA-induced leakage in *Nrp1*^fl/fl^; *Cdh5^CreERT2^* (*Nrp1* iECKO) mice**

Extravasation of circulating 2000 kDa FITC Dextran (pseudocolor) in *Nrp1*^fl/fl^; *Cdh5^CreERT2^* (*Nrp1* iECKO) mice after intradermal injection of VEGFA in the ear dermis. Video corresponds to stills in Figure 1D.

**Video 3: VEGFA-induced leakage in control *Nrp1*^fl/fl^, *Actb* Cre-negative littermate mice**

Extravasation of circulating 2000 kDa FITC Dextran (pseudocolor) in control *Nrp1*^fl/fl^, *Actb* Cre-negative littermate mice after intradermal injection of VEGFA in the ear dermis. Video corresponds to stills in Figure 2C.

**Video 4: VEGFA-induced leakage in *Nrp1*^fl/fl^; *Actb^CreERT2^* (*Nrp1* iKO) mice**

Extravasation of circulating 2000 kDa FITC Dextran (pseudocolor) in *Nrp1*^fl/fl^; *Actb^CreERT2^* (*Nrp1* iKO) mice after intradermal injection of VEGFA in the ear dermis. Video corresponds to stills in Figure 2C.

**Video 5: VEGFA-induced leakage in *Nrp1*^fl/fl^; *Cdh5^CreERT2^* (*Nrp1* iECKO) mice treated with isotype control IgG antibody in the ear dermis**

Extravasation of circulating 2000 kDa FITC Dextran (pseudocolor) in *Nrp1*^fl/fl^; *Cdh5^CreERT2^* (*Nrp1* iECKO) mice pre-treated with an isotype control igG antibody and subsequently challenged with intradermal injection of VEGFA in the ear dermis. Video corresponds to stills in Figure 2G.

**Video 6: VEGFA-induced leakage in VEGFR2 ^Y949F/Y949F^; *Nrp1*^fl/fl^; *Cdh5^CreERT2^* (*Nrp1* iECKO) mice treated with a NRP1-VEGFA blocking antibody in the ear dermis**

Extravasation of circulating 2000 kDa FITC Dextran (pseudocolor) in *Nrp1*^fl/fl^; *Cdh5^CreERT2^* (*Nrp1* iECKO) mice pre-treated with a NRP1-VEGFA blocking antibody and subsequently challenged with intradermal injection of VEGFA in the ear dermis. Video corresponds to stills in Figure 2G.

**Video 7: VEGFA-induced leakage in control VEGFR2 ^Y949F/Y949F^; *Nrp1*^fl/fl^; *Cdh5* Cre-negative littermate mice**

Extravasation of circulating 2000 kDa FITC Dextran (pseudocolor) in VEGFR2 ^Y949F/Y949F^; *Nrp1*^fl/fl^; *Cdh5* Cre-negative littermate mice after intradermal injection of VEGFA in the ear dermis. Video corresponds to stills in Figure 4C.

**Video 8: VEGFA-induced leakage in control VEGFR2 ^Y949F/Y949F^; *Nrp1*^fl/fl^; *Cdh5 ^CreERT2^* mice**

Extravasation of circulating 2000 kDa FITC Dextran (pseudocolor) in VEGFR2 ^Y949F/Y949F^; *Nrp1*^fl/fl^; *Cdh5 ^CreERT2^* mice after intradermal injection of VEGFA in the ear dermis. Video corresponds to stills in Figure 4C.
