# Supplementary material for: Neuropilin-1 controls vascular permeability through juxtacrine regulation of endothelial adherens junctions
Source: Angiogenesis. 2024 Dec 12;28(1):7. doi: 10.1007/s10456-024-09963-3 (PMC11638295; doi:10.1007/s10456-024-09963-3)

Supplemental figure 1

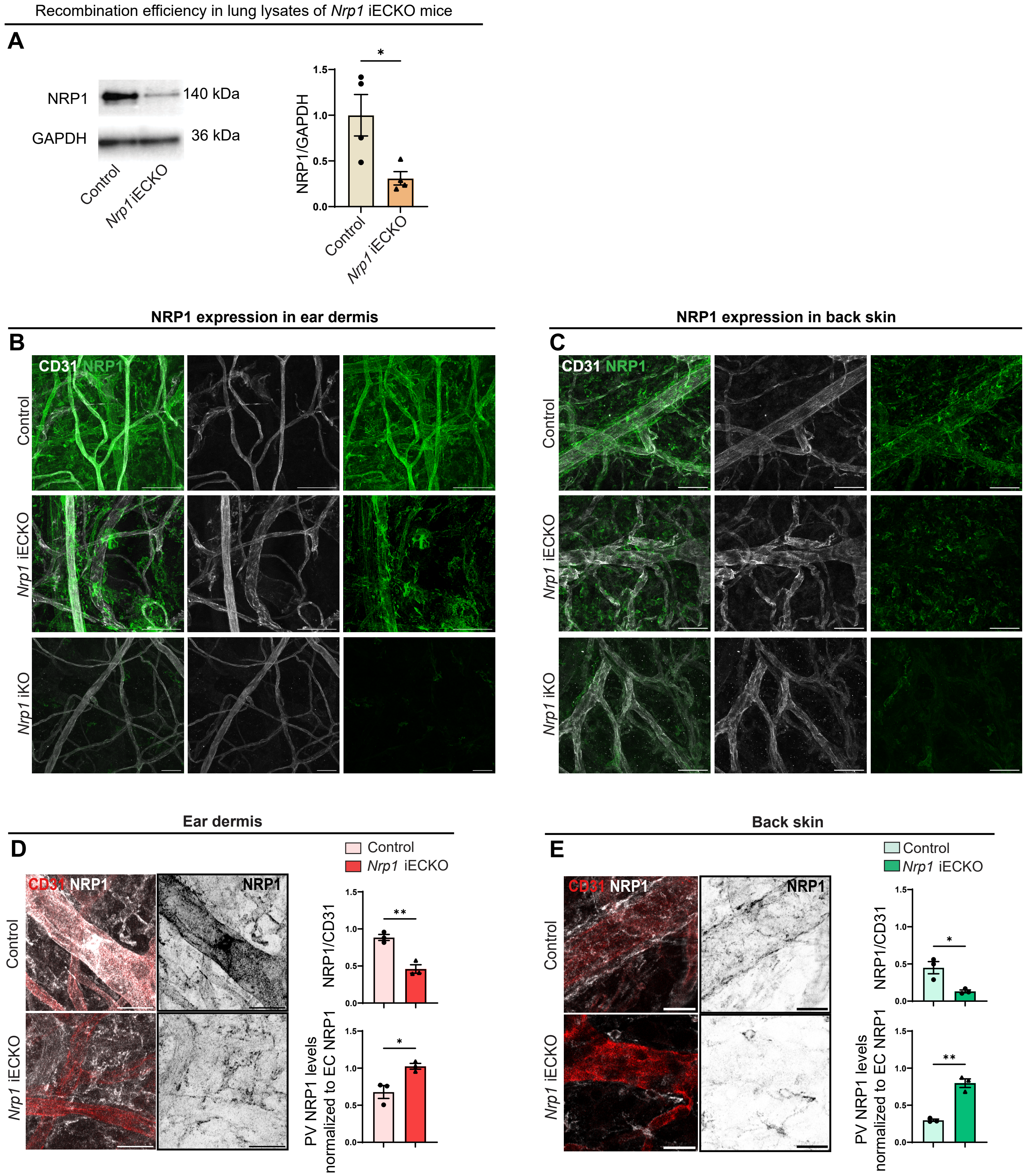

Supplemental figure 2

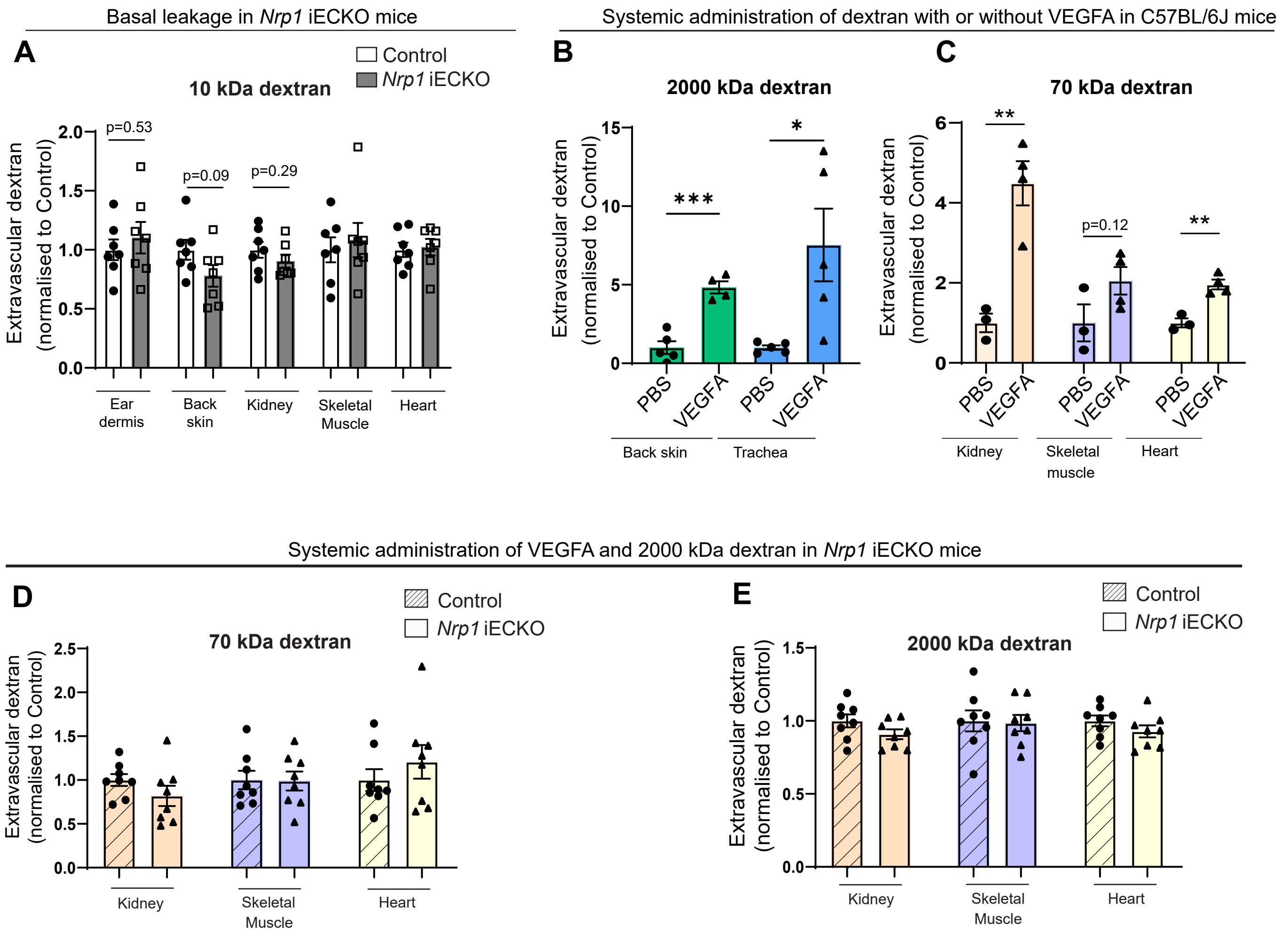

Supplemental figure 3

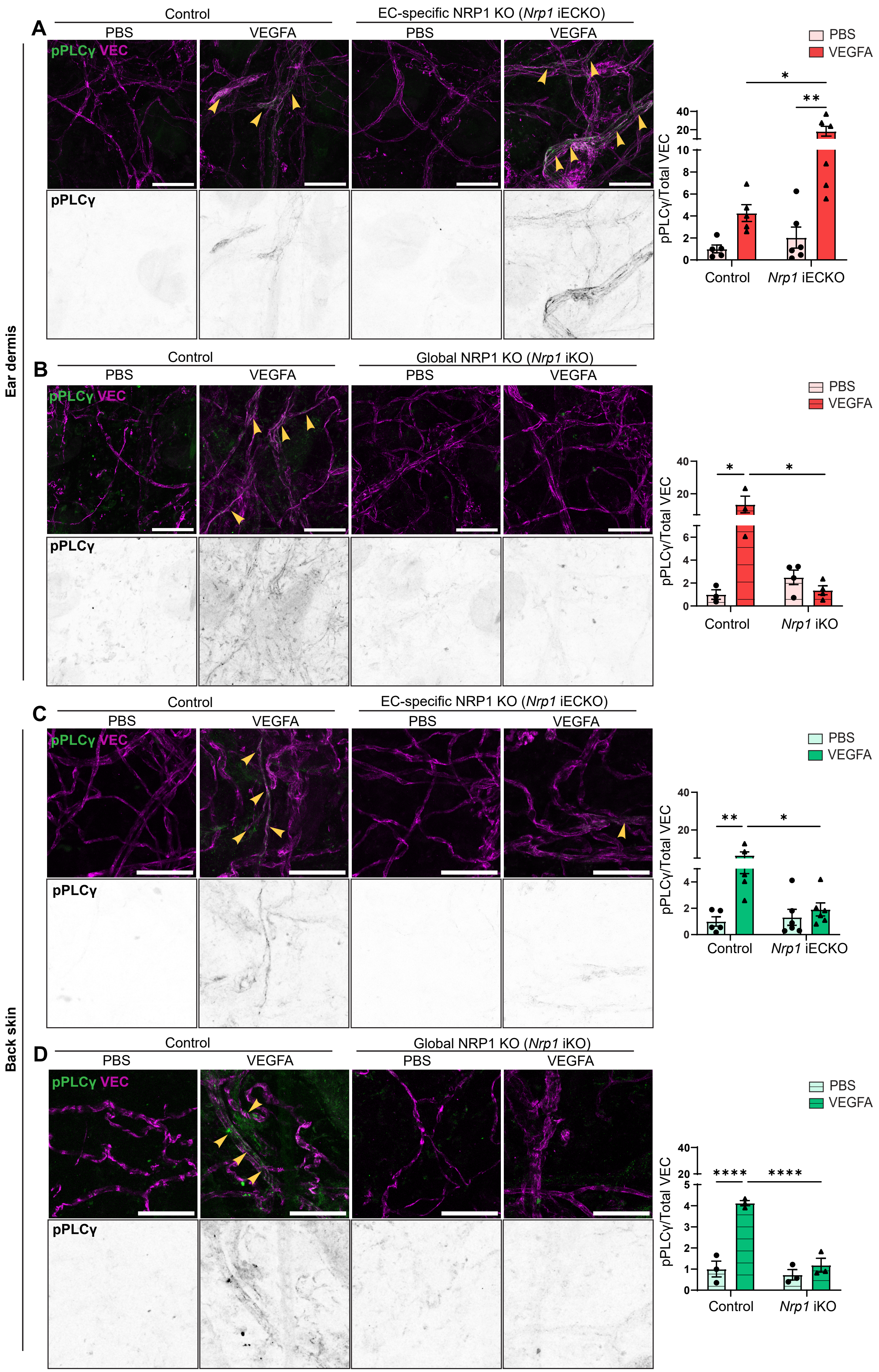

Supplement: Supplementary file 2 — Supplementary file2 (PDF 50828 kb) [file 10456_2024_9963_MOESM2_ESM.pdf]
